# Plasma lipidomic profiling reveals the potential protective role of lipids in cerebral small vessel disease

**DOI:** 10.1186/s12944-026-02891-9

**Published:** 2026-02-12

**Authors:** Chao Wu, Li-Yan Gao, Wei Sun, Ben-Ke Zhao, Ya-Hui Ma, Hai-Qing Song

**Affiliations:** 1 Department of Neurology, Xuanwu Hospital, Capital Medical University, No.45 Changchun Street, Xicheng District, Beijing, 100053 China; 2 Department of Neurology, Qingdao Municipal Hospital, University of Health and Rehabilitation Sciences, Qingdao, China; 3 Department of Neurology, The Affiliated Hospital of Qingdao University, No.16 Jiangsu Road, Qingdao, 266003 China

**Keywords:** Lipidomics, Cerebral Small Vessel Disease, Inflammation, Glycerolipids, Glycerophospholipids

## Abstract

**Background:**

Limited understanding of the pathophysiology of cerebral small vessel disease (CSVD) has hampered the development of effective treatments. Lipidomics offers a promising approach for identifying molecular signatures, clarifying underlying pathogenic mechanisms, and predicting disease severity and progression.

**Methods:**

A total of 1161 participants with lipidomic data from the Alzheimer’s Disease Neuroimaging Initiative (ADNI) database were included and matched to each neuroimaging marker of CSVD separately, including cerebral microbleeds (CMBs, *n* = 578), white matter hyperintensities (WMHs, *n* = 650), lacunes (*n* = 1125), and CSVD burden (*n* = 546). Three complementary classification strategies (class-based grouping, model-based clustering, and individual lipid species) were employed to investigate lipid signatures across various CSVD markers. A multimodel regression framework followed by a series of sensitivity analyses was used to further identify lipid species showing robust associations with each CSVD marker.

**Results:**

A total of 46 lipid classes, 25 lipid clusters, and 749 lipid species were quantified. Individuals with CSVD presented broadly reduced plasma lipid levels, particularly those of glycerophospholipids and glycerolipids. A multimodel regression framework initially screened 32 lipid species associated with the presence or progression of CSVD. Subsequent sensitivity analyses narrowed these to 13 robust species, including phosphatidylcholine, triacylglycerol, phosphatidylethanolamine, alkenyl-phosphatidylethanolamine, and sphingomyelin. Among these, elevated levels of PC(36:4)[+ OH] and TG(56:6)[NL-20:4] were associated with lower odds of CMBs and reduced WMH volumes, respectively, whereas higher levels of PC(15:0_20:3) and SM(d16:1/19:0) were linked to lower odds of lacunes, with all associations consistently observed across baseline, year 1, and year 2.

**Conclusion:**

This study revealed dysregulated lipid metabolism across distinct magnetic resonance imaging phenotypes of CSVD and revealed multiple lipid species that are consistently associated with the presence and progression of these phenotypes, underscoring the potential of lipidomics for the earlier identification and prevention of CSVD and informing future diagnostic development and mechanistic studies.

**Supplementary Information:**

The online version contains supplementary material available at 10.1186/s12944-026-02891-9.

## Background

Cerebral small vessel disease (CSVD) is an umbrella term for a collection of distinct diseases caused by dysfunction of intracranial arterioles, capillaries, and venules, which are commonly related to ageing, hypertension, and genetic factors [1, 2]. Brain magnetic resonance imaging (MRI) is a primary means to detect early manifestations of CSVD. Typical features of CSVD on MRI are cerebral microbleeds (CMBs), white matter hyperintensities (WMHs), small subcortical infarcts or lacunes, visible perivascular spaces, and cerebral atrophy [3]. CSVD is a major global health concern and is responsible for approximately 25% of all ischaemic strokes, the majority of spontaneous intracerebral haemorrhages, and more than 20% of dementia cases [4]. However, the underlying mechanisms of CSVD remain incompletely understood, and no effective treatments exist beyond managing vascular risk factors.

Lipidomics is an emerging discipline at the intersection of lipid biology, analytical technology, and medicine, aiming to construct a comprehensive atlas of the cellular and tissue lipidome [5]. A lipidomic approach enables the simultaneous profiling of hundreds of lipid species, facilitating the identification of key metabolic factors involved in disease pathophysiology. Compared with metabolomics, lipidomics offers greater efficiency and coverage in the identification and quantification of diverse lipid molecular species, particularly those that are highly hydrophobic [6]. Lipidomics has been successfully applied in numerous studies on cardiovascular and neurodegenerative diseases [7, 8], but few studies have investigated its application in CSVD.

Therefore, the present study aimed to systematically evaluate the plasma lipidomic profiles of patients with CSVD compared with those of healthy controls, identify potential associations between dysregulated lipid metabolism and specific MRI phenotypes of CSVD, and identify molecular signatures for future diagnostic or mechanistic applications. Compared with previous studies focusing on conventional lipid measures [9, 10], the present work provides a high-resolution lipidomic perspective, offering novel insights into potential lipid-related mechanisms in CSVD. In addition, given the wide and complex roles of lipids in inflammation [11, 12], associations between plasma lipidomic profiles and CSVD-related inflammatory biomarkers were further examined to provide supplementary insights into lipid‒inflammation interactions in the context of CSVD.

## Methods

### Data source

Data were obtained from the Alzheimer’s Disease Neuroimaging Initiative (ADNI) database (https://adni.loni.usc.edu/). The ADNI is a longitudinal multicentre observational study that provides a comprehensive and widely used collection of longitudinal clinical, imaging, genetic, and other biomarker data.

All participants provided written informed consent, and the institutional review board reviewed and approved the study at each participating site.

### Participants

A total of 1418 participants with both baseline lipidomic profiles and clinical assessments were initially considered. Individuals diagnosed with Alzheimer’s disease (AD) at baseline (*n* = 242) were excluded to reduce potential confounding from AD-related pathology. To address potential data quality issues in the lipidomics dataset, principal component analysis (PCA) was conducted, and 15 extreme outliers falling outside the 99% confidence interval (CI) were removed (Figure S1). The final cohort consisted of 1161 participants.

Given the variability in MRI data availability, associations between lipidomic profiles and each CSVD imaging marker were examined within the respective subset of participants who had valid MRI measurements for that specific marker. This approach maximized statistical power and avoided unnecessary exclusion arising from incomplete imaging across markers. The comparability of demographic and clinical covariates between each imaging-specific subset and the overall analytic cohort was assessed using standardized mean differences (SMDs). An SMD < 0.1 was considered a negligible imbalance, whereas values between 0.1 and 0.2 were interpreted as indicating a mild yet acceptable imbalance [13]. This approach allowed us to evaluate whether covariate patterns identified in the overall cohort could be generalized to imaging-specific subsets.

### Targeted plasma lipidomic profiling

The Baker Heart and Diabetes Institute Metabolomics Laboratory developed a state-of-the-art lipidomics platform, emphasizing precise and reliable measurement of a broad range of lipid species from human plasma. Targeted plasma lipidomic profiling was performed using liquid chromatography–mass spectrometry methodology [14]. Briefly, analysis of plasma extracts was performed on an Agilent 6490 QQQ mass spectrometer with an Agilent 1290 series HPLC system and a ZORBAX eclipse plus C18 column (2.1 × 100 mm 1.8 μm, Agilent) with the thermostat set at 60 °C. Mass spectrometry analysis was performed in positive ion mode with dynamic scheduled multiple reaction monitoring. The detailed lipidomic protocol is described in “ADMC Lipidomics Meikle Lab Longitudinal Data Matrix Methods [ADNI1, GO, 2]”.

A total of 781 lipid species belonging to 47 lipid classes were quantified. Single ion monitoring (SIM) and neutral loss (NL) are two types of measurements for the same triacylglycerol (TG) lipid species, with NL measurements being more specific and sensitive. To minimize redundancy and improve the accuracy of the subsequent modelling analysis, 32 TG[SIM] lipids were excluded, with 749 lipid species from 46 lipid classes retained in the study. Details of all the lipid species and their classifications are presented in Table S1.

### CSVD neuroimaging markers

All subjects were examined using a 1.5/3.0-Tesla MRI scanner, and the procedure was described in a previous report [15]. Details of the parameters are provided on the ADNI website (http://adni.loni.usc.edu/methods/mri-tool/mri-analysis).

### CMBs

CMBs were defined as focal areas of signal void measuring 2–5 mm in diameter (occasionally up to 10 mm) on T2*-weighted or susceptibility-weighted imaging sequences [3]. The number of CMBs was manually rated by trained analysts or radiologists using dedicated software that allows longitudinal tracking of findings. Each finding is catalogued with its location in the MRI scanner coordinate system and assigned a status (“possible,” “definite,” or “rescinded”) on the basis of visual inspection across all available images for the subject. Prior observations are displayed to facilitate longitudinal consistency. Typically, only the CMBs with definite status were selected and counted for analyses in this study. CMBs were recorded as present if there was at least 1 visible CMB.

### WMHs

WMHs were evaluated using both quantitative (WMH volume, WMHV) and semiquantitative (Fazekas score) methods [16]. WMHVs were measured using a Bayesian segmentation approach applied to high-resolution 3D T1 and FLAIR sequences, with specific methods reported in a previous study [17]. WMHV was calculated as the ratio of WMH volume to intracranial volume (ICV) multiplied by 100% (WMHV/ICV × 100%) to standardize for differences in brain size. Fazekas rating scale was used to rate periventricular and deep WMHs separately by two experienced neuroradiologists, with disagreements resolved by a third rater. The weighted kappa (squared) for periventricular WMH Fazekas scores between the two raters was 0.81 (95% CI 0.74–0.88), and that for deep WMH Fazekas scores was 0.79 (95% CI 0.72–0.87), indicating excellent interrater agreement. The total WMH score was then calculated as the sum of the periventricular and deep Fazekas ratings, which ranged from 0 to 6. The severity of WMHs was categorized as 0 (Fazekas scale score, 0), 1 (Fazekas scale score, 1–2), or 2 to 3 (Fazekas scale score, 3–6) [18]. WMHs were recorded as present if either confluent deep WMHs (Fazekas score 2 or 3) or irregular periventricular WMHs extended into the deep white matter (Fazekas score 3) [19].

### Lacunes

Lacunes were defined as a round or ovoid cavity (3–15 mm in diameter), fluid filled (signals similar to cerebrospinal fluid), and consistent with a previous acute small deep brain infarct or haemorrhage in the territory of one perforating arteriole [3]. Lacunes were identified on MRI by physicians who were specifically trained in MRI infarct detection. Lesions ≥ 3 mm were considered, with assessments based on size, location, and imaging characteristics. Images were reviewed using magnified projections to aid interpretation. When multiple images were available across longitudinal sessions, all scans were reviewed to ensure consistency. Interrater reliability among three trained raters was assessed, yielding kappa values between 0.73 and 0.90. Lacunes were recorded as present if there was at least 1 visible lacune.

### CSVD burden

The CSVD burden was quantified using a simple CSVD score based on the presence of CMBs, WMHs (Fazekas score), and the presence of lacunes as described previously [19, 20], which ranged from 0 to 3 points. The severity of CSVD burden was categorized as none (simple CSVD score, 0–1), mild (simple CSVD score, 0–1), or moderate–severe (simple CSVD score, 2–3). CSVD burden was defined as present when the CSVD score was ≥ 1.

### Progression of each CSVD neuroimaging marker

Progression of CMBs, lacunes, and CSVD burden was defined as any increase in their respective counts or scores during follow-up [21]. Considering the potential ceiling effects of the Fazekas score, this study employed WMHV as a continuous quantitative measure to more sensitively capture changes in WMHs over time [22].

### Clinical assessment

The following were determined from baseline clinical evaluation: body mass index (BMI, weight [kilograms]/height [meters] squared), history of hypertension (yes or no), history of diabetes (yes or no), history of hyperlipidaemia (yes or no), history of coronary heart disease (yes or no), history of alcohol abuse (yes or no), history of smoking (yes or no), and use of lipid-modifying agents (LMAs) (yes or no). *Apolipoprotein E* ε4 (APOE *ε*4) carrier status (ε3/*ε*3, ε3/*ε*4, or ε4/*ε*4) was determined from baseline venipuncture as previously described [23]. Routine plasma lipid measures, including triglycerides (TG), low-density lipoprotein cholesterol (LDL-C), and high-density lipoprotein cholesterol (HDL-C), were quantified using Nightingale Health’s nuclear magnetic resonance metabolomics platform.

### Plasma inflammatory biomarkers

The plasma inflammatory protein data were obtained from “Biomarkers Consortium Plasma Proteomics Project RBM multiplex data”. Detailed measurements are described in the Supplemental Methods. On the basis of previous reports [24], the present study focused on CSVD-related inflammatory biomarkers, including biomarkers of systemic inflammation, such as C-reactive protein (CRP), fibrinogen, and cytokines (e.g., interleukin-6 [IL-6], tumour necrosis factor [TNF], tumour necrosis factor receptor 2 [TNFR2] and vascular endothelial growth factor [VEGF]), vascular inflammation markers (e.g., E-selectin, intercellular adhesion molecule-1 [ICAM-1] and vascular cell adhesion molecule-1 [VCAM-1]), and haemostasis-related proteins (e.g., thrombomodulin [TM], von Willebrand factor [vWF], and plasminogen activator inhibitor-1 [PAI-1]). Plasma concentrations of inflammatory biomarkers were measured both at baseline and at one-year follow-up.

### Statistical analyses

Statistical analyses were conducted using R (version 4.3.2). The statistical significance threshold was set at a 2-tailed *P* < 0.05. To account for multiple comparisons, *P* values were adjusted using the Benjamini–Hochberg false discovery rate (FDR) method and reported as FDR *P*.

### Study design

At baseline, the lipidomic dataset was stratified by the presence or severity of each CSVD neuroimaging marker and evaluated across lipid classes, clusters, and species. During the follow-up period, a multimodel analytical framework was applied to identify lipid species associated with both the presence of each CSVD imaging marker at baseline and its subsequent progression over time. These identified lipid species were further filtered by sensitivity analyses and subsequently examined for their associations with CSVD-related inflammatory biomarkers (Figure S2).

### Data transformation and quality control

Lipidomic raw signals were normalized to internal standards to correct for instrument-specific variation, and batch effects between ADNI1 and ADNI GO/2 were mitigated via median centring using the National Institute of Standards Technology 1950 quality control samples. After alignment, lipidomic data, as well as WMHV and inflammatory biomarkers, were log-transformed and z score normalized within each visit (baseline, year 1, and year 2) [25].

### Quantifying the effect of covariates on the lipidome

At baseline, the relative contributions of clinical covariates (i.e., cohort, age, sex, ethnicity, BMI, *APOE Ɛ4* carrier status, hypertension status, diabetes status, hyperlipidaemia status, coronary heart disease status, alcohol abuse status, smoking status, and use of LMAs) to the total variance in the lipidomic data were quantified using linear regression. Normalized lipid intensities were regressed on each covariate individually, and the resulting marginal coefficients of determination (R^2^) were summarized using their median values. Covariates accounting for more than 1% of the total variance were incorporated into the subsequent regression models. The ‘scater’ package in R was used to perform this analysis.

### Model-based clustering

Owing to the high degree of coregulation among lipids within the same structural class, model-based clustering using the ‘mclust’ R package was performed to characterize lipidomic patterns. This approach applies Gaussian finite mixture modelling via the EM algorithm [26]. The optimal model and number of clusters for each solution were determined by considering both the Bayesian information criterion and the number of lipid clusters (LCs), balancing model fit and interpretability. The mean value of the lipids in each cluster was estimated.

### Differential analysis of lipidomic profiles

Three complementary classification strategies (i.e., class-based grouping, model-based clustering, and individual lipid species) were used to investigate the differential distribution of lipidomic profiles across various CSVD imaging markers.

A 2-sample t test was conducted to evaluate the significance of mean differences in lipid intensities between the absence and presence groups of each CSVD imaging marker, performed separately within each lipid cluster, class, and species. Lipid species with *P* < 0.01 and a fold change (FC) > 1.2 or < 0.83 were considered potentially altered. Differences in lipid intensities among subgroups stratified by the severity of WMHs and CSVD burden were assessed using one-way analysis of variance (ANOVA), followed by Tukey’s honest significant difference test, with significance set at *P* < 0.05. Additionally, PCA was performed at both the lipid species and class levels to visualize group separation.

Partial correlation analyses were further conducted to screen the key covariates related to lipid classes or clusters, presented using the ‘qpgraph’ package in R. Spearman correlation analyses were performed to assess the relationships between CSVD subgroups and lipid species, categorized according to fatty acid chain length and degree of unsaturation, across multiple lipid classes.

### Screening for significantly altered lipid species associated with CSVD

A series of regression models were run to identify lipid species significantly associated with each CSVD marker. Baseline lipid species levels were treated as exposure variables, with outcomes including the presence or severity of each CSVD imaging marker at baseline (binary or ordinal logistic regression); baseline WMHV (linear regression); progression of CMBs, lacunes, and CSVD burden (Cox proportional hazards regression); and longitudinal changes in WMHV (linear mixed-effects models). Lipid species were considered notably altered if they showed *P* < 0.05 in both baseline and follow-up analyses. Specifically, for CMBs, lacunes, and overall CSVD burden, concordant results from binary logistic regression at baseline and Cox regression during follow-up were needed, whereas for WMHV, concordance between linear regression at baseline and linear mixed-effects models for longitudinal changes was needed. Covariates previously identified as strongly associated with the lipidome based on R^2^ (i.e., cohort, sex, BMI, hyperlipidaemia, and use of LMAs, as shown in Figure S3), as well as additional variables highly related to CSVD (age, hypertension, and *APOE Ɛ4* carrier status) [22, 27], were the primary adjustment variables in all the regression models.

### Sensitivity analyses

To assess the robustness and reproducibility of the screened lipid species, cross-sectional analyses were repeated at the 1- and 2-year follow-up visits, as these time points had the largest available sample sizes with both lipidomic and CSVD neuroimaging data (Table S2). The lipid species that showed significant associations with CSVD imaging markers at any follow-up time point were identified and retained for downstream analyses. Major atherosclerosis-related risk factors (TG, LDL-C, and HDL-C) were subsequently introduced into the regression models to determine whether the primary associations remained stable after the systemic atherosclerotic burden was accounted for. Correlations between annualized changes in lipid species and longitudinal trajectories of each CSVD imaging marker were further assessed using Cox proportional hazards models or linear mixed-effects models, adjusting for the same covariates as in the primary analyses. Finally, to explore potential effect modification, interaction terms between each lipid species and the covariates (including age, sex, BMI, use of LMAs, and *APOE Ɛ4* carrier status) were incorporated into the models. Interactions with *P* values < 0.1 were considered indicative of possible effect modification, prompting subsequent subgroup analyses.

### Associations between lipidomics and inflammatory biomarkers

Given evidence that systemic inflammation and vascular inflammatory/endothelial dysfunction contribute to the prevalence, severity, and progression of CSVD [24], preliminary cross-sectional analyses were conducted to assess associations between identified lipidomic profiles and CSVD-related inflammatory biomarkers. The inflammatory subset was created by separately merging the baseline (*n* = 319) and 1-year (*n* = 274) lipidomic and inflammatory datasets for downstream analyses. Linear models adjusted for the primary covariates described above were applied, focusing on lipid classes and species significantly associated with CSVD. To evaluate consistency, analyses were performed at both baseline and the 1-year follow-up, and only associations meeting the FDR *P* < 0.05 threshold at both time points were considered significant.

## Results

### Participant characteristics

The overall dataset included 1161 individuals, 749 lipid species across 46 lipid classes, and 25 LCs (Table S1, Figure S4). Most participants were White (92.6%) and male (54.7%), with a mean age of 73.4 (standard deviation [SD] = 6.9) years. At baseline, data for CMBs, WMHs, lacunes, and CSVD burden were available for 578, 650, 1125, and 546 participants, respectively (Table 1). Longitudinal follow-up data were available for 567, 643, 1107, and 482 participants, with mean durations of 4.45 years (range, 0.23–11.98 years), 2.90 years (range, 0.50–6.22 years), 3.97 years (range, 0.21–13.54 years), and 2.41 years (range, 0.37–6.22 years), respectively (Table S2). The numbers of participants with data at years 1 and 2 were as follows: CMBs, 448 and 429; WMHs, 517 and 495; lacunes, 740 and 540; and CSVD burden, 314 and 187. All covariates of interest across each CSVD imaging marker with varying sample sizes showed no or acceptable imbalance compared with the overall participants (SMDs < 0.2). The relative contributions of the covariates to the total variance in the lipidomic profiles were subsequently quantified, and the use of LMAs, BMI, sex, cohort, and hyperlipidaemia were identified as the primary confounding factors (Figure S3).

**Table 1:** Demographic characteristics of participants at baseline

Characteristic	Overall lipidomics sample (*n* = 1161)	Analytic subset (presence^b^), No. (%)							
		CMBs (*n* = 578)		WMHs (*n* = 650)		Lacunes (*n* = 1125)		CSVD burden (*n* = 546)	
		152 (26.3)	SMDs^a^	101 (15.5)	SMDs^a^	84 (7.5)	SMDs^a^	222 (40.7)	SMDs^a^
Age, mean (SD), y	73.4 (6.9)	72.0 (7.0)	0.20	72.2 (7.0)	0.17	73.4 (6.9)	< 0.01	72.1 (7.0)	0.19
Sex, No. (%)									
Male	635 (54.7)	301 (52.1)	0.05	329 (50.6)	0.08	620 (55.1)	0.01	287 (52.6)	0.04
Female	526 (45.3)	277 (47.9)	0.05	321 (49.4)	0.08	505 (44.9)	0.01	259 (47.4)	0.04
Race and ethnicity, No. (%)									
Asian	20 (1.7)	10 (1.7)	< 0.01	10 (1.5)	< 0.01	20 (1.8)	< 0.01	10 (1.8)	< 0.01
Black or African American	50 (4.3)	25 (4.3)	< 0.01	27 (4.2)	< 0.01	50 (4.4)	< 0.01	25 (4.6)	< 0.01
Other or unknown	16 (1.4)	12 (2.1)	0.17	16 (2.5)	0.28	14 (1.2)	0.06	12 (2.2)	0.20
White	1075 (92.6)	531 (91.9)	0.02	597 (91.8)	0.03	1041 (92.5)	< 0.01	499 (91.4)	0.04
*APOE ε/4* carrier status, No. (%)									
* ε3/ε3*	667 (57.5)	341 (59.0)	0.03	382 (58.8)	0.03	647 (57.5)	< 0.01	326 (59.7)	0.04
* ε3/ε*4	404 (34.8)	193 (33.4)	0.03	220 (33.8)	0.02	389 (34.6)	0.01	179 (32.8)	0.04
* ε4/ε*4	90 (7.8)	44 (7.6)	0.01	48 (7.4)	0.02	89 (7.9)	0.01	41 (7.5)	0.01
BMI, mean (SD), kg/m^2^	27.2 (4.8)	27.8 (5.3)	0.12	27.7 (5.2)	0.10	27.2 (4.7)	< 0.01	27.8 (5.1)	0.12
TG, mean (SD), mmol/L	1.2 (0.5)	1.2 (0.5)	< 0.01	1.2 (0.5)	< 0.01	1.2 (0.5)	< 0.01	1.2 (0.5)	< 0.01
LDL-C, mean (SD), mmol/L	1.9 (0.5)	1.9 (0.4)	< 0.01	1.9 (0.4)	< 0.01	1.9 (0.5)	< 0.01	1.9 (0.4)	< 0.01
HDL-C, mean (SD), mmol/L	1.5 (0.4)	1.5 (0.3)	< 0.01	1.5 (0.4)	< 0.01	1.5 (0.4)	< 0.01	1.5 (0.3)	< 0.01
Vascular risk factors, No. (%)									
Hypertension (yes)	506 (43.6)	257 (44.5)	0.02	281 (43.2)	0.01	487 (43.3)	0.01	239 (43.8)	< 0.01
Diabetes (yes)	103 (8.9)	58 (10.0)	0.04	65 (10.0)	0.04	96 (8.5)	0.01	52 (9.5)	0.02
Hyperlipidemia (yes)	534 (46.0)	295 (51.0)	0.10	325 (50.0)	0.08	512 (45.5)	0.01	273 (50.0)	0.08
Coronary heart disease (yes)	54 (4.7)	38 (6.6)	0.07	39 (6.0)	0.06	48 (4.3)	0.01	32 (5.9)	0.06
Smoking (yes)	355 (30.6)	165 (28.5)	0.05	177 (27.2)	0.08	346 (30.8)	< 0.01	156 (28.6)	0.04
Alcohol abuse (yes)	31 (2.7)	13 (2.2)	0.03	14 (2.2)	0.03	30 (2.7)	< 0.01	12 (2.2)	0.03
Use of LMAs (yes)	558 (48.1)	272 (47.1)	0.02	308 (47.4)	0.01	538 (47.8)	0.01	254 (46.5)	0.03

*Abbreviations*: *APOEε4* Apolipoprotein E *ε*4, *BMI* body mass index, *CMBs* cerebral microbleeds, *CSVD* cerebral small vessel disease, *HDL-C* high-density lipoprotein cholesterol, *LDL-C* low-density lipoprotein cholesterol, *LMAs* lipid-modifying agents, *SD* standard deviation, *TG* triglycerides, *WMHs* white matter hyperintensities

^a^Standardized mean differences (SMDs) were used to assess covariate balance between each analytic subset and the overall participants

^b^The presence of each CSVD marker was defined as follows: CMBs counts ≥ 1; either confluent deep WMHs (Fazekas score 2 or 3) or irregular periventricular WMHs extending into the deep white matter (Fazekas score 3); lacunes counts ≥ 1; and CSVD simple scores ≥ 1

### Lipidomic profiles stratified by the presence or absence of CSVD

**Fig. 1:**
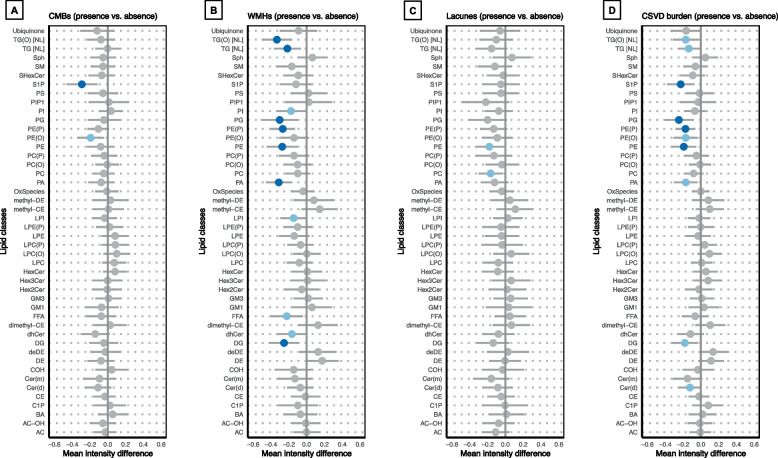
Lipid classes and their mean intensities in individuals with and without CSVD. **A**–**D** correspond to the following CSVD markers: **A** CMBs, **B** WMHs, **C** Lacunes, and **D** CSVD burden. Statistically significant differences in group means (light blue dot: *P* < 0.05; dark blue dot: FDR *P* < 0.05) were compared using a 2-sample t-test. The presence of each CSVD marker was defined as follows: CMBs counts ≥ 1; either confluent deep WMHs (Fazekas score 2 or 3) or irregular periventricular WMHs extending into the deep white matter (Fazekas score 3); lacunes counts ≥ 1; and CSVD simple scores ≥ 1. Abbreviations: CMBs, cerebral microbleeds, CSVD, cerebral small vascular disease, WMHs, white matter hyperintensities

**Fig. 2:**
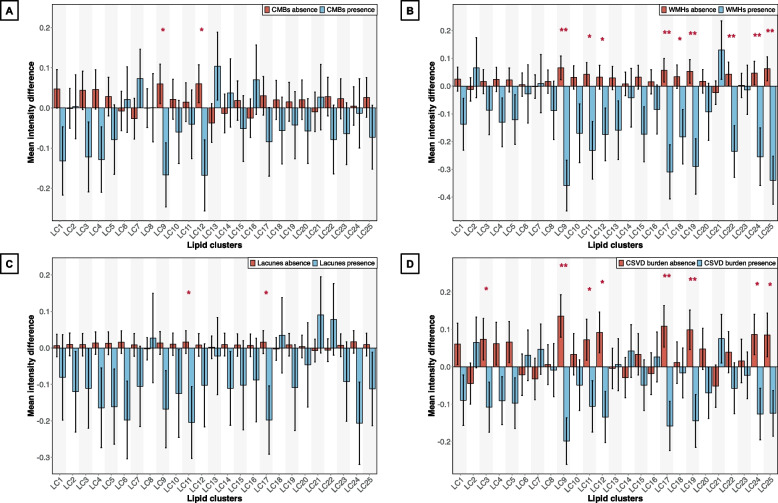
Lipid clusters and their mean intensities in individuals with and without CSVD. **A**–**D** correspond to the following CSVD markers: **A** CMBs, **B** WMHs, **C** Lacunes, and **D** CSVD burden. Statistically significant differences in group means (*P < 0.05; **FDR *P* < 0.05) were compared using a 2-sample t-test. The presence of each CSVD marker was defined as follows: CMBs counts ≥ 1; either confluent deep WMHs (Fazekas score 2 or 3) or irregular periventricular WMHs extending into the deep white matter (Fazekas score 3); lacunes counts ≥ 1; and CSVD simple scores ≥ 1. Lipid species within each cluster were presented in Table S1. Abbreviations: CMBs, cerebral microbleeds; CSVD, cerebral small vascular disease; LC, lipid cluster; WMHs, white matter hyperintensities

Compared with individuals without CSVD neuroimaging markers, those with CSVD markers showed consistently lower normalized lipid intensities across multiple lipid classes, clusters, and individual species. Specifically, at the class level, lipid classes that were significantly reduced (FDR *P* < 0.05) included diacylglycerol (DG), phosphatidic acid (PA), phosphatidylethanolamine (PE), alkenyl-phosphatidylethanolamine (plasmalogen) (PE[P]), phosphatidylglycerol (PG), sphingosine-1-phosphate (S1P), TG, and alkyl-diacylglycerol (TG[O]). Most of these classes included glycerophospholipids (GPs) and glycerolipids (GLs) (Fig. 1). At the cluster level, the mean intensities of LC9 and LC17 were significantly lower in individuals with WMHs and CSVD burden (FDR *P* < 0.05) and decreased separately in those with CMBs or lacunes at the nominal level (*P* < 0.05). These clusters were primarily composed of a mixture of PE and PE(P). LC19, LC22, LC24, and LC25 were also significantly lower in individuals with WMHs (FDR *P* < 0.05), which were primarily composed of DG, free fatty acids (FFAs), TG, and alkyl-diacylglycerol (TG[O]) (Fig. 2 and Table S1). At the species level, multiple lipid species within the PE, PE(P), TG, and TG(O) classes markedly decreased in individuals with WMHs, lacunes, and CSVD burden (*P* < 0.01 and fold change [FC] > 1.2 or < 0.83) (Figure S5 and Table S3). ANOVA results suggested a trend towards decreasing levels of the PA, PE(P), PG, TG(O), and sphingomyelin (SM) classes, as well as the LC9 and LC25 clusters, with increasing severity of WMHs and overall CSVD burden (Figures S6 and S7).

No distinct separation among CSVD subgroups was observed by PCA (Figures S8 and S9). Partial correlation analyses in the overall cohort revealed associations between mean lipid intensities for classes and clusters and multiple covariates (Figures S10 and S11). Consistent with previous findings (Figure S3), cohort, sex, use of LMAs, BMI, and hyperlipidaemia emerged as the primary factors influencing these mean lipid levels. Notably, the mean intensities of most lipid classes and clusters were lower in males, individuals with a higher BMI, those with a history of hyperlipidaemia, and LMA users. Spearman correlation analyses indicated that, across lipid classes stratified by lipid fatty acyl chain length and degree of unsaturation, no separation in correlation patterns was observed between lipid species and the presence of CSVD on the basis of lipid structural features (Figures S12 to S15).

### Significantly altered lipid species associated with CSVD

Using a multimodel regression framework adjusted for cohort, age, sex, *APOE Ɛ4* carrier status, BMI, hypertension, hyperlipidaemia, and use of LMAs, a total of 32 lipid species associated with CSVD were initially screened (Tables S4 and S5). These species spanned several lipid classes, including ceramide (Cer[d], *n* = 1), LPC (*n* = 2), phosphatidylcholine (PC, *n* = 10), alkenyl-phosphatidylcholine (PC[P]), *n* = 1), PE (*n* = 4), alkyl-phosphatidylethanolamine (PE[O], *n* = 1), PE(P) (*n* = 7), SM (*n* = 5), and TG (*n* = 1) (Table S6). After repeated analyses at the 1- and 2-year follow-up intervals (Tables S7 to S10), a set of 13 lipid species showing robust and consistent associations was ultimately identified and retained (Table 2). However, annualized changes in these identified lipid species were not associated with longitudinal changes in CSVD (Table S11). After additional adjustment for TG, LDL-C, and HDL-C, 4 lipid species initially associated with lacunes were attenuated to marginal significance, whereas others remained significant (Table S12). Interestingly, several species within the PC and PE classes share a common acyl chain, 15-methylhexadecanoic acid (15-MHDA), and several identified lipid species associated with lacunes contain odd‑chain fatty acids (OCFAs), mainly C15:0, C17:0, and C19:0.

**Table 2:** Associations between the identified lipid species and each CSVD imaging marker

Outcomes (model)	Plasma levels of identified lipid species across visits (1-SD increase)								
	Baseline		Year 1			Year 2		Baseline	
	*β* (s.e.)	*P* value		*β* (s.e.)	*P* value	*β* (s.e.)	*P* value	*β* (s.e.)	*P* value
CMBs presence or not (MLR)								CMBs progression or not (Cox)	
PC(36:4) [+ OH]	−0.216 (0.101)	0.032	−0.218 (0.108)		0.044	−0.229 (0.108)	0.034	−0.257 (0.081)	0.002
WMHV (MR)								WMHV (MELM)	
TG(56:6) [NL-20:4]	−0.103 (0.038)	0.006	−0.091 (0.040)		0.025	−0.113 (0.041)	0.006	−0.021 (0.006)	0.001
Lacunes presence or not (MLR)								Lacunes progression or not (Cox)	
PC(15:0_20:3)	−0.314 (0.126)	0.013	−0.334 (0.156)		0.032	−0.486 (0.163)	0.003	−0.197 (0.090)	0.028
PC(15:0_20:4)	−0.311 (0.120)	0.010	−0.079 (0.150)		0.601	−0.301 (0.151)	0.047	−0.182 (0.082)	0.026
PC(15-MHDA_18:1)	−0.375 (0.121)	0.002	−0.242 (0.146)		0.099	−0.356 (0.148)	0.017	−0.309 (0.086)	< 0.001
PC(15-MHDA_18:2)	−0.289 (0.123)	0.019	−0.276 (0.145)		0.058	−0.428 (0.152)	0.005	−0.355 (0.087)	< 0.001
PC(15-MHDA_20:4)	−0.276 (0.120)	0.021	−0.201 (0.148)		0.175	−0.372 (0.148)	0.012	−0.249 (0.081)	0.002
PC(15-MHDA_22:6)	−0.304 (0.121)	0.012	−0.320 (0.145)		0.028	−0.217 (0.152)	0.153	−0.262 (0.088)	0.003
PC(17:0_18:1)	−0.366 (0.128)	0.004	−0.253 (0.154)		0.101	−0.333 (0.154)	0.031	−0.228 (0.090)	0.011
PE(15-MHDA_20:4)	−0.310 (0.117)	0.008	−0.047 (0.146)		0.750	−0.382 (0.153)	0.013	−0.162 (0.081)	0.047
PE(P-17:0/22:6) (a)	−0.271 (0.117)	0.020	−0.288 (0.145)		0.047	−0.145 (0.146)	0.323	−0.202 (0.083)	0.015
SM(37:1)	−0.295 (0.126)	0.020	−0.304 (0.152)		0.046	−0.300 (0.154)	0.051	−0.214 (0.088)	0.015
SM(d16:1/19:0)	−0.283 (0.118)	0.017	−0.292 (0.143)		0.041	−0.302 (0.145)	0.037	−0.273 (0.083)	0.001

*Abbreviations*: *APOE Ɛ4* Apolipoprotein E ε4, *BMI* body mass index, *CMBs* cerebral microbleeds, *Cox* Cox proportional hazards regression model, *CSVD* cerebral small vessel disease, *LMAs* lipid-modifying agents, *MELM* mixed-effects linear model, *15-MHDA* 15-methylhexadecanoic acid, *MLR* multiple logistic regression model, *MR* multiple linear regression model, *PC* phosphatidylcholine, *PE* phosphatidylethanolamine, *PE(P)* alkenyl-phosphatidylethanolamine (plasmalogen), *SE* standard error, *SM* sphingomyelin, *TG* triacylglycerol, *WMHV* white matter hyperintensity volume

All regression models were adjusted for cohort, age, sex, *APOE Ɛ4* carrier status, BMI, hypertension, hyperlipidemia, and use of LMAs

### Subgroup analyses

The interaction effects by age, sex, BMI, use of LMAs, and *APOE Ɛ4* carrier status are presented in Table S13 and Table S14. In the cross-sectional analysis, significant interactions were found for sex with lipid species related to CMBs and for the use of LMAs with lipid species associated with WMHV. Specifically, in males, lower levels of PC(36:4) [+ OH] were associated with higher odds of CMBs (odds ratio [OR] = 0.68 [95% CI, 0.52–0.89], *P* = 0.005, *P* for interactio*n* = 0.045), whereas in females, the association was in the opposite direction but did not reach significance. Among individuals who were not users of LMAs, the level of TG (56:6) [NL-20:4] was negatively associated with WMHV (*β* = −0.197, *P* < 0.001, *P* for interactio*n* = 0.021) in the linear regression model, whereas no significant association was observed in those who used LMAs (Table S13).

In the longitudinal analysis, significant interactions were found for age and BMI with lipid species related to lacunes and for use of LMAs with lipid species associated with WMHV. Among individuals aged ≤ 70 years, lower baseline levels of PE (P-17:0/22:6) (a) (hazard ratio [HR] = 0.66 [95% CI, 0.48–0.92], *P* = 0.013; *P* for interactio*n* = 0.080) and SM (37:1) (HR = 0.57 [95% CI, 0.38–0.84]; *P* = 0.004; *P* for interactio*n* = 0.044) were significantly associated with a greater risk of incident lacune progression. In those with a BMI > 25 kg/m^2^, baseline levels of PC(15:0_20:3) (HR = 0.75 [95% CI, 0.60–0.95], *P* = 0.015, *P* for interactio*n* = 0.063) and PC(15-MHDA_18:2) (HR = 0.65 [95% CI, 0.52–0.81], *P* < 0.001, *P* for interactio*n* = 0.062) were negatively associated with incident lacune progression, while PC(15-MHDA_18:2) was also negatively associated with a BMI ≤ 25 kg/m^2^. Among individuals who did not use LMAs, elevated baseline TG (56:6)[NL-20:4] was linked to a slower increase in WMHV over time (*β* = −0.194; *P* < 0.001; *P* for interactio*n* = 0.047) in the linear mixed-effects model, which is consistent with the cross-sectional findings (Table S14).

### Lipidomic associations with CSVD-related inflammatory biomarkers

The baseline characteristics of the inflammatory subset are presented in Table S15 and show no or acceptable imbalance compared with those of the overall cohort (SMDs < 0.2). At the class level, the mean intensities of PE and PG were positively associated with VEGF levels at the threshold of FDR *P* < 0.05 (Figure S16). In contrast, the mean intensity of PC was inversely associated with TNFR2 and VCAM-1, whereas Cer(d) was positively associated with fibrinogen, and PE(O) was negatively associated with TNFR2; however, these correlations were only nominally significant (*P* < 0.05). At the species level, the level of PE (15-MHDA_20:4) was positively associated with VEGF at the threshold of FDR *P* < 0.05 (Figure S17). In contrast, PC(15-MHDA_22:6) levels correlated negatively with CRP and TNFR2 levels, and PE(P-17:0/22:6) (a) was inversely associated with TNFR2, although these relationships were only nominally significant (*P* < 0.05).

## Discussion

The present study characterized plasma lipidomic profiles linked to CSVD, providing a broader view of potential metabolic alterations. In the present study, individuals with CSVD imaging marker burden exhibited reduced mean intensities of multiple lipid classes, mainly from GP and GL. Further analyses revealed 13 specific lipid species, including PC (*n* = 8), PE (*n* = 1), PE (P) (*n* = 1), SMs (*n* = 2), and TG (*n* = 1), whose baseline levels were significantly negatively correlated with both the presence and progression of CSVD. Additionally, this study preliminarily explored the associations between these significantly altered lipid classes and species and CSVD-related inflammatory markers, suggesting a potential interplay between disrupted lipid metabolism and inflammatory processes in CSVD pathogenesis.

Integrating cross-sectional results from both lipid class and lipid cluster levels revealed reduced mean intensities across multiple lipid classes, including Cer(d), FFA, DG, PA, PC, PE, PE(O), PE(P), PG, S1P, TG, and TG(O). This observed widespread downregulation suggested that lower levels of these lipid classes might be associated with CSVD pathogenesis, supported by existing evidence: a prior metabolomics study found that decreased levels of GP, sphingolipids (SP), and sterol lipids were associated with greater CSVD burden [28]; consistently, a large-scale study (*n* = 118,021) from the UK Biobank identified inverse associations between circulating levels of fatty acids, phospholipids, and triglycerides and WMHV [29]. Of note, prior studies have widely reported associations between multiple Cer(d) and SM species and CSVD [28, 30, 31]. In contrast, in the baseline analyses, the mean intensities of these two classes showed only limited associations with CSVD. Although six lipid species related to lacunes (one Cer(d) and five SMs) passed the initial screening, only two SMs, SM(37:1) and SM(d16:1/19:0), remained significant after the full evaluation. This discrepancy may stem from the more stringent screening strategy, which required consistency across cross-sectional and longitudinal analyses and incorporated more extensive confounder adjustment.

Notably, the levels of several phospholipids (i.e., PC, PE, and PE[P]) significantly decreased across multiple CSVD imaging markers. These alterations suggested that disrupted phospholipid metabolism might be involved in the pathophysiological processes of CSVD. Disruption of the blood–brain barrier (BBB) and chronic neuroinflammation are hallmark pathological features and are considered early events in the occurrence and progression of CSVD [32, 33]. Prior evidence has indicated that plasma phospholipid levels tend to be associated with the risk of cardiovascular diseases [34], especially given the dual role of plasma phospholipids in regulating endothelial function, with some species enhancing endothelial barrier integrity and others increasing endothelial permeability [35, 36]. In the present study, higher levels of PC(36:4) [+ OH], an oxidized phospholipid (OxPL), were associated with lower odds of CMBs, and the primary characteristics of CMBs were endothelial dysfunction and BBB integrity loss [37, 38]. Notably, several full-length OxPLs exhibit potential pro-survival and anti-inflammatory effects on endothelial cells [39]. Therefore, a plausible hypothesis was that PC(36:4) [+ OH] might strengthen BBB endothelial barrier function, which could in turn contribute to a lower risk of CMB occurrence and progression. A previous study also reported markedly reduced serum levels of several PC and LPC species in individuals with peripheral arterial disease and coronary artery disease [40]. Consistent with these findings, this study revealed inverse associations between multiple PC species and lacunes. However, the biological mechanisms linking reduced PC levels to lacunar pathology remain to be elucidated.

In this study, higher levels of several PC and PE species containing 15-MHDA, a monomethyl branched-chain fatty acid (mmBCFA), were associated with lower odds and progression of lacunes. Previous studies have suggested potential neuroprotective effects of mmBCFAs, such as reducing infarct volume and cerebral oedema, decreasing BBB permeability, and exerting anti-inflammatory effects [41, 42]. However, the biofunctions of phospholipid species incorporating mmBCFAs remain largely unexplored. The findings of this study suggested that lower levels of specific mmBCFA-containing lipids were associated with the presence and progression of lacunes. Odd-chain fatty acids (OCFAs) are present in very low amounts, accounting for nearly 1% of the total fatty acids in human plasma. Their origins are multifaceted and include dietary intake, gut microbiota metabolism, and endogenous synthesis [43]. Interestingly, several lipid species associated with lacunes identified in this study were OCFAs (mainly C15:0, C17:0, and C19:0). Given the rigorous targeted lipidomics platform used, these signals likely reflect true biological variation rather than analytical artefacts [14]. Previous studies have suggested that OCFAs, particularly C15:0 and C17:0, may be negatively associated with cardiovascular and metabolic diseases [44–46], which supported the possibility of a similar association between OCFAs and lacunes in the present study. However, the absence of dietary information in the ADNI cohort (e.g., food-frequency questionnaire) limits the ability to determine whether the observed associations were driven by differences in dietary exposures. Therefore, further studies are needed to clarify whether OCFAs have causal effects on CSVD pathology.

Notably, no annualized changes in retained lipid species were associated with the progression of any CSVD imaging markers, which may be explained by several factors, such as the limited sensitivity of annualized lipid changes in capturing meaningful biological alterations and potential variability in lipid measurements over time. Another possibility is that baseline lipid levels may have a more sustained influence on disease development than long-term fluctuations, implying that the absolute concentrations at baseline, rather than subsequent temporal changes, were more informative for assessing their relevance to CSVD.

Subgroup analyses indicated that lipid-lowering therapy may modulate the negative associations between identified lipid species and CSVD imaging markers. Clinical application of lipid-lowering therapy in patients with CSVD remains controversial. Prospective studies investigating the effects of lipid-lowering interventions on CSVD have yielded inconsistent results [47, 48]. In contrast, several cross-sectional studies reported that higher LDL-C levels were associated with slower WMH progression [49, 50], despite LDL-C being a primary target of lipid-lowering therapy. In the present study, the links between specific lipid species and CSVD markers were more pronounced in individuals who did not receive lipid-lowering therapy, indicating potential effect modification by treatment status. Overall, lipidomic profiling offers a relatively high-resolution perspective that could help clarify some of the existing uncertainties surrounding lipid-lowering interventions in patients with CSVD.

VEGF is the most specific growth factor of endothelial cells and is known as the main inducer of angiogenesis [51]. VEGF plays vital roles in coordinating neurovascular homeostasis. Pathological elevation of VEGF promotes the breakdown of the BBB [52, 53], which was deemed the mechanism involved in the progression of CSVD. In the present study, increased mean intensities of PE and PG and increased levels of PE(15-MHDA_20:4) were negatively associated with WMHV, lacunes, and CSVD burden but positively associated with increased levels of VEGF, which seemed contradictory. A possible explanation is that under physiological conditions, these lipid species may enhance endothelial function and promote microvascular repair via VEGF-mediated signalling [54], whereas a reduction in their levels could attenuate this potential effect. Unfortunately, owing to the limited sample size of inflammatory marker data and their imperfect alignment with CSVD imaging markers, lipidomics–VEGF–CSVD cascade associations could not be explored. Future studies with larger, well-matched cohorts are warranted to clarify these mechanisms.

### Study strengths and limitations

The present study had several strengths. First, compared with conventional metabolomics, high-throughput lipidomics enables a broader and more precise quantification of lipid species. Second, multilevel analyses (i.e., lipid classes, lipid clusters, and individual species) provide a comprehensive view of lipid metabolic alterations in patients with CSVD. Third, key covariates related to lipidomic profiles were carefully identified and rigorously adjusted for in regression models, and a multiple time-point approach was applied to reduce bias from single measurements, thereby enhancing the robustness and credibility of the findings in this study.

Several limitations of the present study should be acknowledged. First, the ADNI cohort was not specifically designed for CSVD research. However, its inclusion and exclusion criteria and standardized MRI protocols were similar to those used in dedicated CSVD cohorts [55], making it a reasonably suitable cohort for observational CSVD analyses. Second​​, although all the data originated from the ADNI cohort, systematic differences in lipidomic profiles persisted across subcohorts (ADNI-1, GO, and 2) because of technical variations in sample handling, storage duration, and analytical platforms. While subcohort differences were adjusted for in the analyses, residual confounding cannot be excluded. External validation in independent cohorts is therefore essential to confirm the generalizability of these findings. Third​​, the identification of altered lipid species in cross-sectional and longitudinal analyses used a nominal *P* < 0.05 threshold rather than an FDR *P* < 0.05. This might increase false-positive risks from multiple testing. Nonetheless, given the exploratory nature of this study, using FDR correction might have been overly stringent and risked overlooking biologically meaningful associations. In addition, lipid selection in this study was based on consistent associations across both cross-sectional and longitudinal analyses and multiple time points, leading to the relative robustness of these findings. Finally, the majority of participants were White, the follow-up period was insufficient, longitudinal data were not available, and the data concerning CSVD-related imaging markers were incomplete. Consequently, these findings should be validated in larger, multiethnic cohorts with longer follow-up periods and in better-phenotyped CSVD cohorts.

## Conclusions

The present study revealed signatures of plasma lipid metabolism across specific MRI phenotypes of CSVD and identified multiple lipid species showing consistent associations with the presence and progression of CMBs, WMHs, and lacunes. These findings suggest that lipidomic profiling might help characterize metabolic heterogeneity among individuals with CSVD, increase the understanding of the variable responses to lipid-related preventive or therapeutic strategies observed in clinical practice, support earlier identification of patients at greater risk of CSVD progression, and provide a metabolic framework to inform future diagnostic and mechanistic investigations. Future studies should focus on whether these associations are robust and causal and investigate the underlying mechanisms between lipid metabolism and CSVD pathophysiology.

## Supplementary Information

Supplementary Material 1.

Supplementary Material 2.

Supplementary Material 3.

## Data Availability

All data from this study could be obtained from the ADNI database.

## Abbreviations

AD: Alzheimer’s disease
ADNI: Alzheimer’s disease neuroimaging initiative
APOE Ɛ4: Apolipoprotein E ε4
ANOVA: Analysis of variance
BBB: Blood-brain barrier
BMI: Body mass index
Cer(d): Ceramide
CI: Confidence interval
CMBs: Cerebral microbleeds
Cox: Cox proportional hazards regression model
CRP: C-reactive protein
CSVD: Cerebral small vessel disease
DG: Diacylglycerol
FC: Fold change
FDR: False discovery rate
FFA: Free fatty acid
GL: Glycerolipids
GP: Glycerophospholipids
HR: Hazard ratio
ICAM-1: Intercellular adhesion molecule-1
HDL-C: High-density lipoprotein cholesterol
ICV: Intracranial volume
IL-6: Interleukin-6
LC: Lipid cluster
LDL-C: Low-density lipoprotein cholesterol
LMAs: Lipid-modifying agents
MELM: Mixed-effects linear model
15-MHDA: 15-Methylhexadecanoic acid
MLR: Multiple logistic regression model
MRI: Magnetic resonance imaging
NL: Neutral loss
OCFAs: Odd‑chain fatty acids
OR: Odds ratio
PA: Phosphatidic acid
PAI-1: Plasminogen activator inhibitor-1
PC: Phosphatidylcholine
PCA: Principal component analysis
PC(P): Alkenyl-phosphatidylcholine
PE: Phosphatidylethanolamine
PE(O): Alkyl-phosphatidylethanolamine
PE(P): Alkenyl-phosphatidylethanolamine (plasmalogen)
PG: Phosphatidylglycerol
S1P: Sphingosine-1-phosphate
SD: Standard deviation
SIM: Single ion monitoring
SM: Sphingomyelin
SMDs: Standardized mean differences
SP: Sphingolipid
TG: Triacylglycerol
TG(O): Alkyl-diacylglycerol
TM: Thrombomodulin
TNF: Tumor necrosis factor
TNFR2: Tumor necrosis factor receptor 2
VCAM-1: Vascular cell adhesion molecule-1
VEGF: Vascular endothelial growth factor
vWF: Von Willebrand factor
WMHs: White matter hyperintensities
WMHV: White matter hyperintensity volume
