# Supplementary material for: Plasma lipidomic profiling reveals the potential protective role of lipids in cerebral small vessel disease
Source: Lipids Health Dis. 2026 Feb 12;25:83. doi: 10.1186/s12944-026-02891-9 (PMC12997876; doi:10.1186/s12944-026-02891-9)
Supplement: Supplementary file 1 — Supplementary Material 1. [file 12944_2026_2891_MOESM1_ESM.docx]

**Plasma lipidomic profiling reveals the potential protective role of lipids in cerebral small vessel diseas~~e~~**

Chao Wu, MD, PhD^1^, Li-Yan Gao, MD, PhD^2^, Wei Sun, MD, PhD^1^, Ben-Ke Zhao, MD, PhD^1^, Ya-Hui Ma, MD, PhD^3,*^ , Prof. Hai-Qing Song^1,*^; for the Alzheimer’s Disease Neuroimaging Initiative^#^

^1^ Department of Neurology, Xuanwu Hospital, Capital Medical University, Beijing, China;

^2^ Department of Neurology, Qingdao Municipal Hospital, University of Health and Rehabilitation Sciences, Qingdao, China;

^3^ Department of Neurology, The Affiliated Hospital of Qingdao University, Qingdao, China;

^#^ Data used in preparation of this article were obtained from the Alzheimer’s Disease Neuroimaging Initiative (ADNI) database (http://adni.loni.usc.edu). As such, the investigators within the ADNI contributed to the design and implementation of ADNI and/or provided data but did not participate in the analysis or writing of this report. A complete listing of ADNI investigators can be found at: <https://adni.loni.usc.edu/wp-content/uploads/how_to_apply/ADNI_Acknowledgement_List.pdf>.

* Correspondence to:

Prof. Hai-Qing Song, Department of Neurology, Xuanwu Hospital, Capital Medical University, No.45 Changchun Street, Xicheng District, Beijing 100053, China. Tel: +86

Dr. Ya-Hui Ma, Department of Neurology, The Affiliated Hospital of Qingdao University, No.16 Jiangsu Road, Qingdao 266003, China. Tel: +86 17853280115; E-mail: yahuiweixiao@163.com (YH. Ma).

‬**Supplemental Figures**

**Figure S1**. Principal component analysis of all 1176 lipidomic samples at baseline.

**Figure S2.** Flowchart of Study design.

**Figure S3.** Contributing factors affecting the variance of the lipidomic dataset.

**Figure S4.** Performances of Gaussian mixture models for the clustering of the molecular lipids.

**Figure S5.** Lipid species and their mean levels in individuals with and without CSVD.

**Figure S6.** Lipid classes at baseline for 3 groups stratified by WMH/CSVD severity.

**Figure S7.** Lipid clusters at baseline for 3 groups stratified by WMH/CSVD severity.

**Figure S8.** Principal component analysis of lipid species labelled by absence and presence groups across each CSVD neuroimaging marker.

**Figure S9.** Principal component analysis of lipid classes labelled by absence and presence groups across each CSVD neuroimaging marker.

**Figure S10.** Partial correlation analysis of lipid classes and clinical variables at baseline.

**Figure S11.** Partial correlation analysis of lipid clusters and clinical variables at baseline.

**Figure S12.** Spearman correlations between CMBs subgroups and lipid species grouped by fatty acid composition.

**Figure S13.** Spearman correlations between WMHs subgroups and lipid species grouped by fatty acid composition.

**Figure S14.** Spearman correlations between lacunes subgroups and lipid species grouped by fatty acid composition.

**Figure S15.** Spearman correlations between CSVD burden subgroups and lipid species grouped by fatty acid composition.

**Figure S16.** Associations between identified lipid classes and CSVD-related inflammatory biomarkers.

**Figure S17.** Associations between identified lipid species and CSVD-related inflammatory biomarkers.

**Figure S1. Principal component analysis of all 1176 lipidomic samples at baseline.**

The ellipse denoted a 99% confidence region. The variance explained by principal components 1 and 2 was shown. The red dots outside the 99% confidence ellipse indicated participants who would be excluded from subsequent analyses (n = 15).

**Figure S2.** **Flowchart of Study design.**

Abbreviations: AD, Alzheimer's Disease; CMBs, cerebral microbleeds; Cox, Cox proportional hazards regression model; CSVD, cerebral small vessel disease; FC, fold change; LCs, lipid clusters; LMAs, lipid-modifying agents; MELM, mixed-effects linear model; MLR, multiple logistic regression model; MR, multiple linear regression model; PCA, principal component analysis; QC, quality control; WMHs, white matter hyperintensities.

**Figure S3. Contributing factors affecting the variance of the lipidomic dataset.**

The percentage of explained variance of all factors across the lipid samples was shown. The variables that explained more than 1% variance in lipidomics profiles were the use of LMAs, BMI, sex, cohort, and hyperlipidemia, which were considered key covariates in the regression models.

Abbreviations: *APOE Ɛ4*, *Apolipoprotein E ε4*; BMI, body mass index; LMAs, lipid-modifying agents.

**Figure S4. Performances of Gaussian mixture models for the clustering of the molecular lipids.**

Several spherical and diagonal models, *i.e*., ‘EII’, ‘VII’, ‘EEI’, ‘EVI’, ‘VEI’, and ‘VVI’, were evaluated by EM algorithm coded in the ‘mclust’ package. The optimal model was automatically selected based on the Bayesian information criterion (BIC).

**Figure S5. Lipid species and their mean levels in individuals with and without CSVD.**

Volcano plots showed the differential plasma lipid species between individuals with and without CSVD. Red dots indicated lipid species with *P* < 0.01 and FC > 1.2; blue dots indicated lipid species with *P* < 0.01 and FC < 0.83; gray dots indicate non-significant species.

Abbreviations: CMBs, cerebral microbleeds; CSVD, cerebral small vascular disease; FC, fold change; WMHs, white matter hyperintensities.

**Figure S6. Lipid classes at baseline for 3 groups stratified by WMH/CSVD severity.**

*Statistically significant difference of the group means as determined by analysis of variance and post hoc Tukey’s honestly significant difference (HSD). The severity of WMHs was categorized as 0 (Fazekas scale score, 0), 1 (Fazekas scale score, 1–2), and 2 to 3 (Fazekas scale score, 3–6). The severity of CSVD burden was categorized as none (simple CSVD score, 0–1), mild (simple CSVD score, 0–1), and moderate–severe (simple CSVD score, 2–3).

Abbreviations: CSVD, cerebral small vascular disease; WMHs, white matter hyperintensities.

**Figure S7. Lipid clusters at baseline for 3 groups stratified by WMH/CSVD severity.**

*Statistically significant difference of the group means as determined by analysis of variance and post hoc Tukey’s honestly significant difference (HSD). The severity of WMHs was categorized as 0 (Fazekas scale score, 0), 1 (Fazekas scale score, 1–2), and 2 to 3 (Fazekas scale score, 3–6). The severity of CSVD burden was categorized as none (simple CSVD score, 0–1), mild (simple CSVD score, 0–1), and moderate–severe (simple CSVD score, 2–3).

Abbreviations: CSVD, cerebral small vascular disease; LC, lipid cluster; WMHs, white matter hyperintensities.

**Figure S8. Principal component analysis of lipid species labelled by absence and presence groups across each CSVD neuroimaging marker.**

The ellipse denotes 95% confidence region. Variance of the samples along the principal components 1 and 2 denotes are shown.

Abbreviations: CMBs, cerebral microbleeds; CSVD, cerebral small vascular disease; FC, fold change; WMHs, white matter hyperintensities.

**Figure S9.** **Principal component analysis of lipid classes labelled by absence and presence groups across each CSVD neuroimaging marker.**

The ellipse denotes 95% confidence region. Variance of the samples along the principal components 1 and 2 denotes are shown.

Abbreviations: CMBs, cerebral microbleeds; CSVD, cerebral small vascular disease; FC, fold change; WMHs, white matter hyperintensities.

**Figure S10.** **Partial correlation analysis of lipid classes and clinical variables at baseline.**

About 10,000 tests for non-rejection were performed for each pair of variables included in the correlation matrix. All the spurious correlations/associations (nonrejection rates < 0.5) were removed. Spearman’s rank correlation coefficients were estimated, with only those reaching *P* < 0.05 displayed in this figure. Red denoted positive and blue denoted negative correlations.

Abbreviations: *APOE Ɛ4*, *Apolipoprotein E ε4*, BMI, body mass index; LMAs, lipid-modifying agents.

**Figure S11. Partial correlation analysis of lipid clusters and clinical variables at baseline.**

About 10,000 tests for non-rejection were performed for each pair of variables included in the correlation matrix. All the spurious correlations/associations (nonrejection rates < 0.5) were removed. Spearman’s rank correlation coefficients were estimated, with only those reaching *P* < 0.05 displayed in this figure. Red denoted positive and blue denoted negative correlations.

Abbreviations: *APOE Ɛ4*, *Apolipoprotein E ε4*, BMI, body mass index; LMAs, lipid-modifying agents.

**Figure S12. Spearman correlations between CMBs subgroups and lipid species grouped by fatty acid composition.**

Spearman’s coefficient is color-coded with red as a positive and blue as a negative correlation between the lipid species levels in the presence vs. absence of CMBs.

**Figure S13. Spearman correlations between WMHs subgroups and lipid species grouped by fatty acid composition.**

Spearman’s coefficient is color-coded with red as a positive and blue as a negative correlation between the lipid species levels in the presence vs. absence of WMHs.

**Figure S14. Spearman correlations between lacunes subgroups and lipid species grouped by fatty acid composition.**

Spearman’s coefficient is color-coded with red as a positive and blue as a negative correlation between the lipid species levels in the presence vs. absence of lacunes.

**Figure S15. Spearman correlations between CSVD burden subgroups and lipid species grouped by fatty acid composition.**

Spearman’s coefficient is color-coded with red as a positive and blue as a negative correlation between the lipid species levels in the presence vs. absence of CSVD burden.

**Figure S16. Associations between identified lipid classes and CSVD-related inflammatory biomarkers.**

Statistically significant differences (**P*＜0.05; **FDR *P*＜0.05) of relationships between lipid classes and inflammatory biomarkers. Models were adjusted for age, sex, *APOE Ɛ4* carrier status, BMI, hypertension, hyperlipidemia, and use of LMAs.

Abbreviations: *APOE Ɛ4,* *Apolipoprotein E ε4*; BMI, body mass index; CRP, C-reactive protein; Fib, fibrinogen; IL-6, interleukin-6; ICAM-1, intercellular adhesion molecule-1; LMAs, lipid-modifying agents; PAI-1, plasminogen activator inhibitor-1; TNF-α, tumor necrosis factor alpha; TNFR2, tumor necrosis factor receptor 2; VEGF, vascular endothelial growth factor; VCAM-1, vascular cell adhesion molecule-1; TM, thrombomodulin; vWF, von Willebrand factor.

**Figure S17. Associations between identified lipid species and CSVD-related inflammatory biomarkers.**

Statistically significant differences (**P*＜0.05; **FDR *P*＜0.05) of relationships between lipid species and inflammatory biomarkers. Models were adjusted for age, sex, *APOE Ɛ4* carrier status, BMI, hypertension, hyperlipidemia, and use of LMAs.

Abbreviations: *APOE Ɛ4,* *Apolipoprotein E ε4*; BMI, body mass index; CRP, C-reactive protein; Fib, fibrinogen; IL-6, interleukin-6; ICAM-1, intercellular adhesion molecule-1; LMAs, lipid-modifying agents; PAI-1, plasminogen activator inhibitor-1; TNF-α, tumor necrosis factor alpha; TNFR2, tumor necrosis factor receptor 2; VEGF, vascular endothelial growth factor; VCAM-1, vascular cell adhesion molecule-1; TM, thrombomodulin; vWF, von Willebrand factor.
