# Supplementary material for: Plasma lipidomic profiling reveals the potential protective role of lipids in cerebral small vessel disease
Source: Lipids Health Dis. 2026 Feb 12;25:83. doi: 10.1186/s12944-026-02891-9 (PMC12997876; doi:10.1186/s12944-026-02891-9)
Supplement: Supplementary file 2 — Supplementary Material 2. [file 12944_2026_2891_MOESM2_ESM.docx]

**Plasma lipidomic profiling reveals the potential protective role of lipids in cerebral small vessel disease**

Chao Wu, MD, PhD^1^, Li-Yan Gao, MD, PhD^2^, Wei Sun, MD, PhD^1^, Ben-Ke Zhao, MD, PhD^1^, Ya-Hui Ma, MD, PhD^3,*^ , Prof. Hai-Qing Song^1,*^; for the Alzheimer’s Disease Neuroimaging Initiative^#^

^1^ Department of Neurology, Xuanwu Hospital, Capital Medical University, Beijing, China;

^2^ Department of Neurology, Qingdao Municipal Hospital, University of Health and Rehabilitation Sciences, Qingdao, China;

^3^ Department of Neurology, The Affiliated Hospital of Qingdao University, Qingdao, China;

^#^ Data used in preparation of this article were obtained from the Alzheimer’s Disease Neuroimaging Initiative (ADNI) database (http://adni.loni.usc.edu). As such, the investigators within the ADNI contributed to the design and implementation of ADNI and/or provided data but did not participate in the analysis or writing of this report. A complete listing of ADNI investigators can be found at: <https://adni.loni.usc.edu/wp-content/uploads/how_to_apply/ADNI_Acknowledgement_List.pdf>.

* Correspondence to:

Prof. Hai-Qing Song, Department of Neurology, Xuanwu Hospital, Capital Medical University, No.45 Changchun Street, Xicheng District, Beijing 100053, China. Tel: +86

Dr. Ya-Hui Ma, Department of Neurology, The Affiliated Hospital of Qingdao University, No.16 Jiangsu Road, Qingdao 266003, China. Tel: +86 17853280115; E-mail: yahuiweixiao@163.com (YH. Ma).‬‬

**Supplemental Methods**

**Plasma inflammatory biomarkers**

Plasma samples were sent frozen to Rules-Based Medicine (RBM) in Austin, TX, USA. There they were assayed without additional freeze-thaw cycles. RBM conducted a multiplexed immunoassay via their human multi-analyte profile (MAP). A complete listing of the human MAP biomarker panel is available at <http://www.rulesbasedmedicine.com>. This panel included proteins previously associated with inflammation, cancer, cardiovascular disease, and lipid metabolism. All biomarker assays were performed in duplicate, and measurements with >5% variation between replicates were excluded. Values below the detection limit (LOW) were handled based on frequency: analytes with >50% LOW values were excluded, while others were imputed as half the least detectable dose (LDD). Outliers (>3 standard deviations from the mean) were removed.
